# APT1-derived depalmitoylation of CD36 alleviates diabetes-induced lipotoxicity in podocytes

**DOI:** 10.7150/ijbs.109220

**Published:** 2025-06-09

**Authors:** Juan Wang, Jijia Hu, Hongtu Hu, Qian Guan, Zijing Zhu, Qian Yang, Guohua Ding

**Affiliations:** 1 Division of Nephrology, Renmin Hospital of Wuhan University, Wuhan, Hubei, China.; 2 Nephrology and Urology Research Institute of Wuhan University, Wuhan, Hubei, China.

**Keywords:** Podocyte, APT1, CD36, Lipotoxicity, Diabetic kidney disease

## Abstract

Cluster of Differentiation 36 (CD36), also known as scavenger receptor B2, plays a critical role in controlling podocyte lipid metabolism, mediating the onset and progression of diabetic kidney disease (DKD). However, the post-translational regulation of CD36 and its exact role in lipid transport within podocytes remain unclear. In this study, we elucidate the mechanism by which acyl-protein thioesterase 1 (APT1) depalmitoylates CD36 in podocytes. We reveal that APT1 interacts with CD36 and reduces its palmitoylation at Cys466 specifically, thereby promoting its trafficking from the plasma membrane to lysosomes for degradation. Diabetes-induced downregulation of APT1 redirects palmitoylated CD36 into the recycling pathway. Consequently, enhanced lipid uptake in podocytes leads to lipotoxicity. Conversely, APT1 overexpression mitigates lipid accumulation by enhancing lysosomal degradation and reducing plasma membrane-associated CD36. Our findings indicate that diabetes-induced APT1 deficiency promotes palmitoylated CD36 enrichment on plasma membranes through decreased APT1 expression, driving lipid overload and podocyte injury.

## Introduction

Diabetic kidney disease (DKD) is the primary cause of end-stage renal disease (ESRD) worldwide 1. The main manifestations of DKD include increased urinary albumin excretion and a progressive decline in the glomerular filtration rate, which are closely associated with podocyte foot process effacement and podocyte shedding or loss 2, 3. In diabetes, impaired glucose metabolism is often accompanied by dysregulated lipid metabolism, characterized by elevated levels of cholesterol, triglycerides, free fatty acids (FFAs), and other lipids in the circulation and peripheral tissues 4. The lipid nephrotoxicity hypothesis, proposed by Moorhead, highlights the critical role of renal lipid accumulation in DKD progression 5.

Podocytes are particularly susceptible to lipid accumulation, a central mechanism driving DKD advancement 6. Our previous studies explored podocyte cholesterol metabolism, revealing that abnormal cholesterol trafficking in diabetic podocytes could lead to intracellular cholesterol accumulation and subsequent injury 7-10. However, the molecular mechanisms underlying diabetes-induced disruption of fatty acid metabolism in podocytes remain insufficiently understood. FFAs are transported into cells via Cluster of Differentiation 36 (CD36), a fatty acid transporter protein 11. CD36 functions as a key regulator of lipid homeostasis and energy availability, playing an important role in the cellular uptake of FFAs 12, 13. Growing evidence has confirmed that altered and abnormally distributed CD36 expression leads to ectopic renal lipid deposition, contributing to lipotoxic kidney injury 14. Notably, increased CD36 expression has been observed in renal biopsies of patients with DKD and the glomeruli of diabetic animal models 15. Beyond the transcriptional activation of CD36 in diabetes, such as C/EBP, PXR, and LXR 14,16, the posttranslational regulatory mechanisms have garnered increasing attention 17. The post-translational modifications of CD36 include palmitoylation, glycosylation, ubiquitination, and phosphorylation 18. The glycosylation of CD36 plays a crucial role in modulating its structure and function, and is particularly critical for the recognition of oxidized low-density lipoprotein (oxLDL) and involvement in cholesterol metabolism 19. Phosphorylation is likely correlated with ligand binding and may influence the interaction of CD36 with thrombospondin 14. The polyubiquitination pathway targets CD36 for proteasomal degradation 18. Importantly, emerging studies have emphasized that palmitoylation modification is involved in lipid metabolism and is closely associated with the membrane localization of proteins 20.

Acyl-protein thioesterase 1 (APT1) is a depalmitoylase 21 and a redox sensor that responds to oxidative stress 22. Recent investigations have underscored that APT1 takes a key role in depalmitoylation modification 23, 24, and is involved in cellular lipid metabolism regulation 25. APT1 has been shown to regulate CD36 palmitoylation in adipocytes 25. However, whether APT1 contributes to diabetes-induced podocyte lipotoxicity by affecting the posttranscriptional regulation of CD36 remains unclear. This study identified a novel role for APT1 in modulating cellular fatty acid metabolism by altering the subcellular localization and function of CD36 through palmitoylation regulation. These findings suggest that APT1 may represent a potential therapeutic target for podocyte injury in DKD.

## Results

### Renal morphology and lipid metabolism in the glomeruli of db/db mice

**Figure 1:**
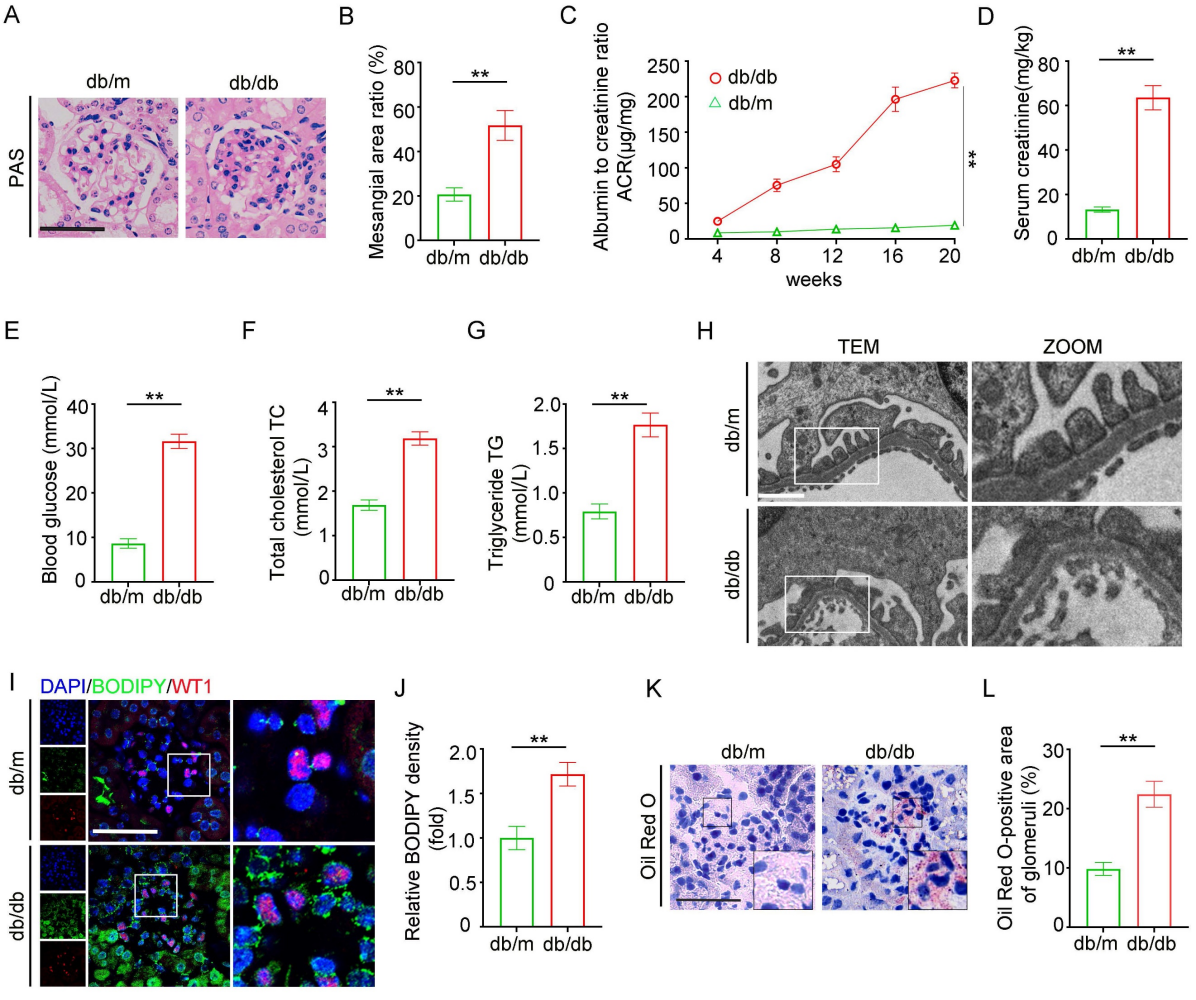
** Construction of db/db model to evaluate changes in renal pathology lipid metabolism.** Two groups of twenty-week-old mice were analyzed: db/m (n=6) and db/db (n=6). A-B. Representative PAS staining of glomeruli in each group, and the semiquantitative analysis of mesangial area ratio. (**<0.01, scale bar: 50 μm). C-G. Biochemical examination: (C) UACR, (D) serum creatinine, (E) blood glucose, (F) serum cholesterol, (G) serum triglycerides. (**<0.01). H. Representative transmission electron microscopy (TEM) images of podocyte foot processes and glomerular basement membrane (GBM) in each group. (**<0.01, scale bar: 10 μm). I-J. Representative immunofluorescence double staining of BODIPY and WT1 in the glomeruli of db/m and db/db mice, and semiquantitative analysis. (**<0.01, scale bar:50μm). K-L. Oil Red O staining of lipid droplets (LDs) in the glomeruli of db/m and db/db mice, and semiquantitative analysis. (**<0.01, scale bar: 40 μm).

An animal model of DKD was established using db/db mice, which exhibited mesangial cell proliferation and matrix accumulation in the glomeruli (Figure 1A-B), increased urine albumin creatinine ratio (UACR) and serum creatinine levels, and elevated blood glucose compared to db/m mice (Figure 1C-E). In addition, db/db mice showed significant dyslipidemia, with total cholesterol (TC) and triglycerides (TG) higher than those in the db/m group (Figure 1F-G). Histological examination revealed extensive podocyte foot process effacement under transmission electron microscopy (TEM) in db/db mice, aligning with the pathological features of diabetic nephropathy (Figure 1H). Fluorescent dye BODIPY 493/503, co-labeled with Wilms' Tumor 1 (WT1, a podocyte marker), indicated marked lipid accumulation in glomeruli and WT1-positive podocytes (Figure 1I-J). Oil Red O staining revealed elevated neutral lipid deposition in db/db glomeruli (Figure 1K-L). These results collectively suggest that glomerular lipid accumulation contributes to podocyte injury and renal dysfunction in the DKD mouse model.

### Profiling of gene expression involved in CD36 palmitoylation in the glomeruli of db/db mice

**Figure 2:**
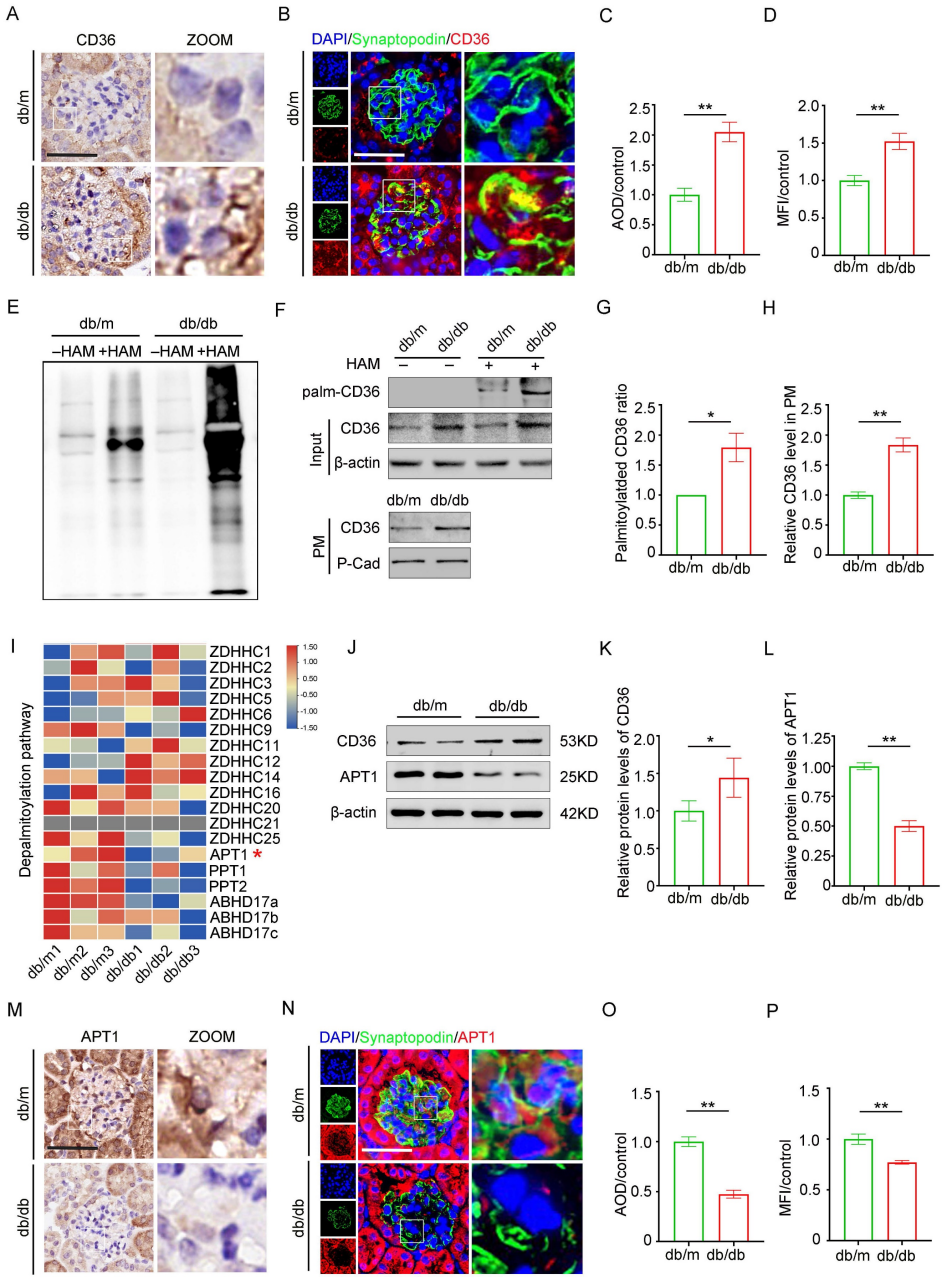
** Protein expression of CD36, APT1 and palmitoylation of total Protein in the glomeruli of mice with Diabetic Nephropathy.** A, C. Representative immunohistochemical labeling of CD36 in kidney sections of control and DKD mice, and semiquantitative analysis. (**<0.01, scale bar: 50 μm). B, D. Immunofluorescence staining of CD36 (red) with synaptopodin (a marker of podocyte, green), and DAPI (nucleus, blue) in the glomeruli of control and DKD mice, and semiquantitative analysis. (**<0.01, scale bar: 50 μm). E. Palmitoylation level of total protein in the glomeruli of control and db/db mice by ABE method and Western blot. F-H. The level of palmitoylated CD36 in glomeruli by IP-ABE and Western blot methods, and representative Western blot of membrane-associated CD36 in control and DKD mice, and semiquantitative analysis. (*<0.05, **<0.01). I. Heatmap of palmitoyl-acyltransferases (PATs) and acyl-protein thioesterases (APTs). J-L. The protein expression of APT1 and CD36 in the glomeruli of control and DKD mice, and semiquantitative analysis. (*<0.05, **<0.01). M, O. Representative immunohistochemical labeling of APT1 in kidney sections of control and DKD mice, and semiquantitative analysis. (**<0.01, scale bar: 50 μm). N, P. Immunofluorescence staining of APT1 (red) with synaptopodin (green) and DAPI (blue) in the glomeruli of control and DKD mice, and semiquantitative analysis. (**<0.01, scale bar: 50 μm).

CD36 is essential for fatty acid uptake in podocytes. Previous studies have demonstrated a positive correlation between CD36 expression and lipid accumulation in the glomeruli of patients with DKD compared to control kidney biopsy samples 15. Consistent with previous research, immunohistochemistry (Figure 2A, 2C), and immunofluorescence co-staining with the podocyte marker synaptopodin (Figure 2B, 2D) revealed upregulated CD36 expression in the podocytes and db/db glomeruli, in contrast to db/m mice. Since CD36 is regulated by multiple post-translational modifications, we assessed the effects of specific inhibitors and confirmed that palmitoylation is vital for its subcellular localization (Supplement 1A-B). Glomeruli isolated from db/db mice exhibited a significant increase in total palmitoylated protein levels, suggesting enhanced palmitoylation activity (Figure 2E). Furthermore, db/db mice displayed elevated palmitoylated CD36 levels, an increased ratio of palmitoylated to total CD36, and higher membrane-associated CD36 expression (Figure 2F-H). To investigate the underlying mechanism of CD36 palmitoylation dysregulation, RNA-sequencing data from db/db and db/m mouse glomeruli were analyzed, focusing on genes encoding palmitoyl-acyltransferases (PATs) and acyl-protein thioesterases (APTs) (Figure 2I). Among these, APT1 was notably downregulated in the db/db glomeruli. Although previous studies in adipocytes suggest that APT1 regulates CD36 palmitoylation 25, the exact mechanism in kidney podocytes remains unclear. Here, downregulation of APT1 in podocytes and glomeruli from db/db mice was further confirmed through Western blotting (Figure 2J-L), immunohistochemistry (Figure 2M, 2O), and immunofluorescence co-staining with synaptopodin (Figure 2N, 2P), compared with control mice.

### Interaction of CD36 and APT1 in podocytes under the high glucose stimulation

**Figure 3:**
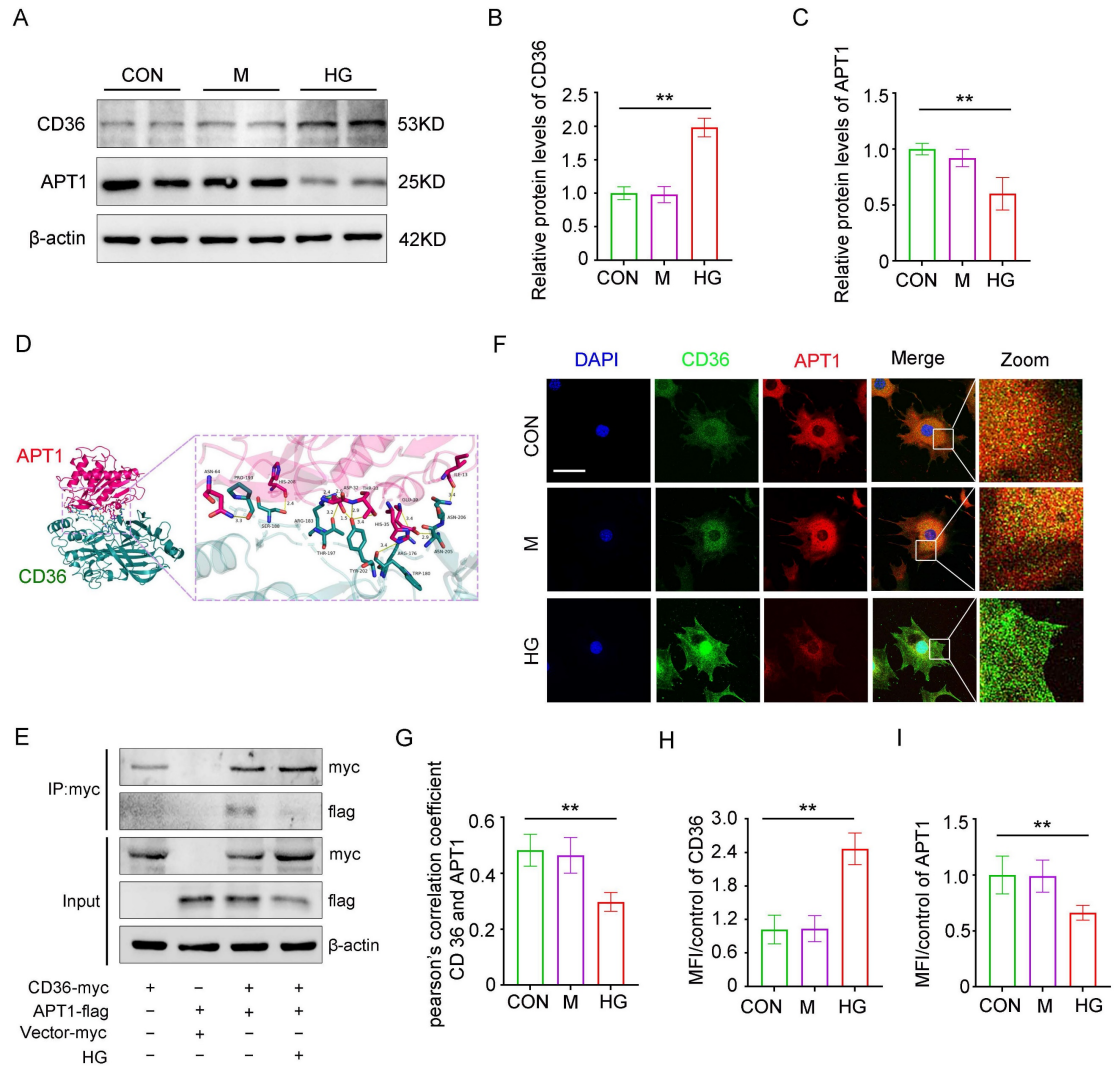
** Interaction between APT1 and CD36 *in vitro* under high glucose stimulation.** Podocytes were divided into three groups and exposed for 24 hours under the corresponding stimulation: Control (CON), 5 mM glucose; Mannitol (M), 5 mM glucose + 25 mM mannitol; HG, 30 mM glucose. A-C. Representative Western blot of APT1 and CD36 in HPCs, and semiquantitative analysis. (**<0.01). D. The direct binding sites between APT1 and CD36 were predicted by AlphaFold database. E. CO-IP of myc-CD36 and flag-APT1 in podocytes immunoprecipitated by anti-myc antibody. F-I. Representative confocal microscopy image of APT1 and CD36 immunofluorescence double staining, Pearson's correlation coefficient, and semiquantitative analysis. (**<0.01, scale bar: 40 μm)

Consistent with *in vivo* results, *in vitro* studies revealed that APT1 was downregulated while CD36 was upregulated in podocytes under high glucose (HG) stimulation (Figure 3A-C). To exclude the influence of hypertonic conditions, a mannitol control group was included. Structural prediction analysis using AlphaFold revealed a putative direct binding interface between CD36 and APT1 (Figure 3D). Next, this interaction was confirmed through co-immunoprecipitation and confocal fluorescence imaging. Podocytes were then transfected with myc-tagged wild-type CD36 plasmid, flag-tagged wild-type APT1 plasmid, or control plasmids, with or without HG stimulation. Immunoblotting of cell lysates using a myc antibody showed that APT1 binds to CD36 under both HG and control conditions (Figure 3E). However, in the HG state, the myc-CD36 level was increased, and the binding of APT1 was significantly reduced compared to the normal glucose group. Reverse validation using a flag antibody confirmed these findings (Supplement 1C). Additionally, confocal-based fluorescence imaging demonstrated reduced APT1 fluorescence intensity, and enhanced CD36 fluorescence intensity in podocytes with HG stimulation (Figure 3F, H-I). However, decreased co-localization of APT1 and CD36 in HG-stimulated podocytes was observed (Figure 3F-G). These results suggest that APT1 interacts with CD36, and that HG stimulation leads to APT1 downregulation and diminished binding to CD36. However, the precise mechanism by which APT1 influences CD36 subcellular localization requires further investigation.

### Effect of APT1-mediated depalmitoylation on the intracellular trafficking of CD36

**Figure 4:**
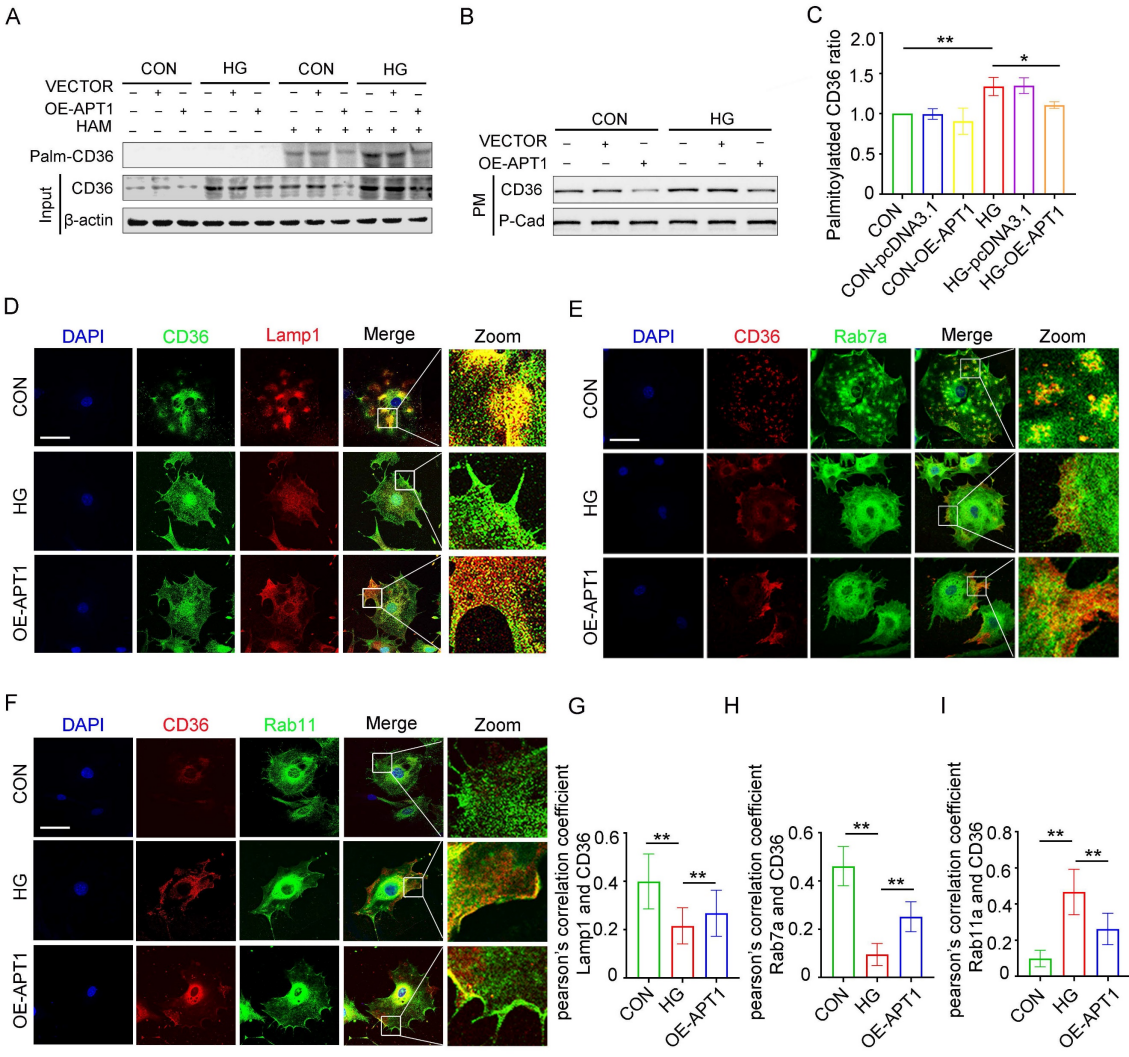
** The changes in the palmitoylated CD36 ratio and the CD36 degradation-recycling pathway in HPCs under HG.** A-C. Podocytes were transfected with empty vector or APT1 plasmid, then incubated with or without 30 mM high glucose for 24 hours. The level of palmitoylated CD36 in podocytes by IP-ABE and Western blot methods (A); the amount of membrane-associated CD36 in podocytes (B) in each group, and semiquantitative analysis (C). (*<0.05, **<0.01). D-F. Confocal microscopy images of CD36 and lamp1 (D), CD36 and Rab7a (E), CD36 and Rab11a (F) in each group: CON, HG, OE-APT1. (scale bar: 40 μm). G-I. The respective Pearsonʼs correlation coefficients. (**<0.01).

The mechanism of intracellular CD36 trafficking was investigated by altering APT1 expression in podocytes. Transfection with an APT1 overexpression plasmid *in vitro* revealed that APT1 upregulation reduced the palmitoylated CD36 ratio and decreased CD36 levels on the plasma membrane, partially reversing the changes induced by HG conditions (Figure 4 A-C, Supplement 2A-C). This observation led to a deeper exploration of the regulatory mechanisms governing CD36 subcellular transport. CD36 was co-stained with protein markers for lysosomes (Lamp1, Figure 4D), late endosomes (Rab7a, Figure 4E), and recycling endosomes (Rab11a, Figure 4F). Imaging indicated decreased co-localization of CD36 with Rab7a and Lamp1, alongside increased co-localization with Rab11a, suggesting that under HG stimulation more CD36 is retained in recycling endosomes and less is degraded by the endosome-lysosome pathway. Pearson's correlation quantified these co-localizations, with APT1 overexpression reversing the observed effects (Figure 4G-I). Further co-immunoprecipitation experiments demonstrated that CD36 co-precipitates with Rab11 in HG conditions (Supplement 2D-E), indicating Rab11's role in CD36 membrane trafficking within podocytes.

To further elucidate how APT1 regulates the endocytic recycling and lysosomal degradation of CD36, immunoprecipitation of CD36 followed by ubiquitination analysis revealed significantly enhanced CD36 ubiquitination in human podocytes (HPCs) transfected with the APT1 overexpression plasmid compared to controls (Supplement 2F), while palmitoylation was concurrently reduced. These findings demonstrate an inverse relationship between CD36 palmitoylation and ubiquitination. Collectively, these outcomes provide the first evidence that APT1 modulates CD36 palmitoylation under HG stimulation and influences its degradation-recycling pathways, resulting in increased accumulation of palmitoylated CD36 on the podocyte membrane in HG conditions.

### Effects of mutating different palmitoylation sites on CD36 subcellular localization and function

**Figure 5:**
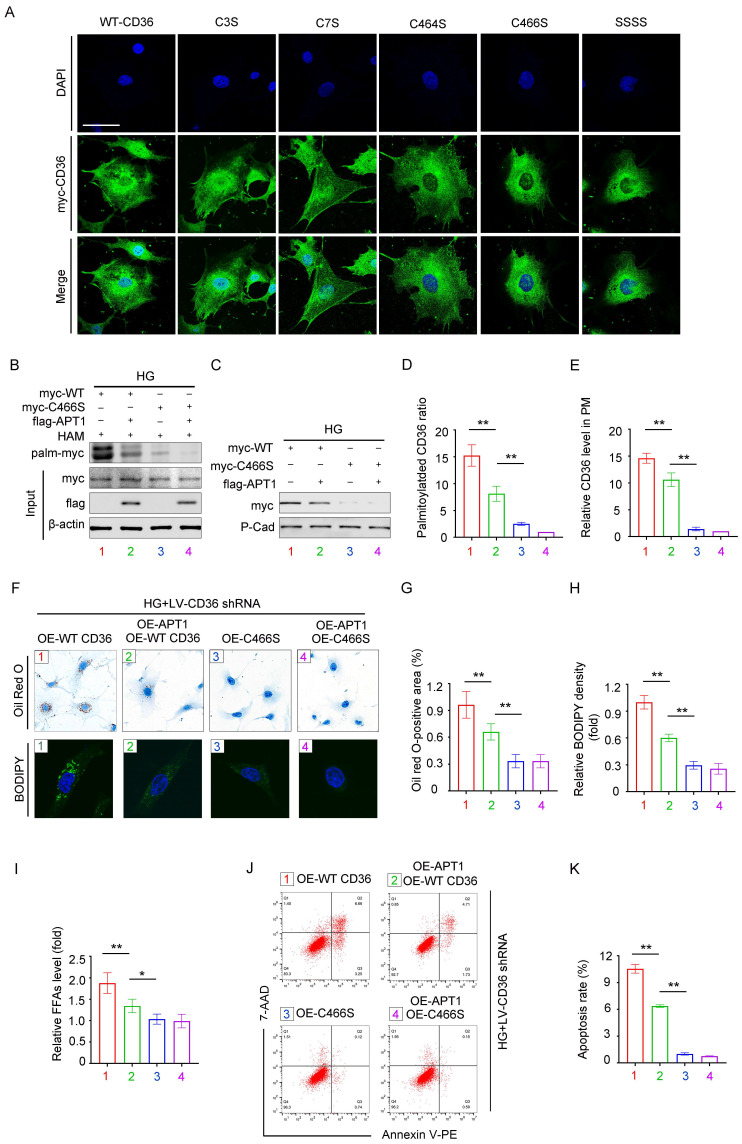
** Mutation at CD36 palmitoylation sites affects its subcellular localization and podocyte injury.** A. Representative confocal microscopy images of myc-CD36 staining in HPCs transfected with site-directed mutant CD36 plasmids or wild-type CD36 plasmid. (scale bar: 40 μm). B-E. Podocytes were divided into four groups, (1) myc-WT CD36 overexpression plasmid transfection, (2) myc-WT CD36 overexpression plasmid and flag-APT1 overexpression plasmid co-transfection, (3) myc-C466S CD36 overexpression plasmid transfection, (4) myc-C466S CD36 overexpression plasmid and flag-APT1 overexpression plasmid co-transfection, and then exposed to 30 mM HG for 24 hours. Representative image of palmitoylated CD36 (B), membrane-associated CD36 (C), and semiquantitative analysis (D, E). (**<0.01). F-H. First endogenous CD36-KO podocyte cell line is constructed with LV-shCD36, then divided into four groups according to the transfection scheme in B. Representative microscopy images of lipid droplets by Oil Red O (scale bar: 40 μm) and BODIPY staining (scale bar: 20 μm) in each group (F), and semiquantitative analysis (G, H). (**<0.01). I. Cellular free fatty acids semiquantitative analysis in each group as described in F. (*<0.05, **<0.01). J-K. Flow cytometry analysis of podocyte apoptosis and semiquantitative analysis in each group as described in F. (**<0.01).

Previous studies identified four palmitoylation sites on CD36: Cys3, Cys7, Cys464, and Cys466. To investigate which site affects the CD36 membrane trafficking mostly under high glucose conditions, we constructed mutant CD36 plasmids: myc-C3S, myc-C7S, myc-C464S, myc-C466S, and myc-SSSS. Podocytes were transfected with either wild-type (WT) or mutant CD36 plasmids. CD36 trafficking was visualized by confocal microscopy (Figure 5A). To validate this observation, HPCs were then transfected with myc-WT CD36 or myc-C466S overexpression plasmids, with or without the flag-APT1 plasmid, under HG conditions. The acyl-biotinyl exchange (ABE) assay revealed that the C466S mutation substantially reduced palmitoylation and membrane translocation (Figure 5B-E), consistent with confocal microscopy findings. Notably, the Cys466 mutation significantly reduced CD36 presence on the plasma membrane. Then, CD36 knockout (KO) podocyte cell line was generated using lentivirus to eliminate endogenous CD36 interference. Lipid accumulation, assessed by Oil Red O staining and BODIPY 493/503, was significantly lower in the myc-C466S group, regardless of the presence of flag-APT1. In contrast, lipid accumulation was reduced in the myc-WT CD36 group with flag-APT1, compared to the myc-WT CD36 group without it (Figure 5F-H). A similar pattern was observed for intracellular FFA content (Figure 5I). Furthermore, apoptosis measured by flow cytometry showed significantly lower apoptosis rates in both the C466S and myc-WT CD36 group with flag-APT1 compared to the myc-WT CD36 group without flag-APT1 (Figure 5J-5K). Thus, these results underscore the critical role of the Cys466 site in facilitating CD36 membrane trafficking, among the four palmitoylation sites.

### APT1 inhibitor ML348 reverses the effects of APT1 plasmid in HPCs

**Figure 6:**
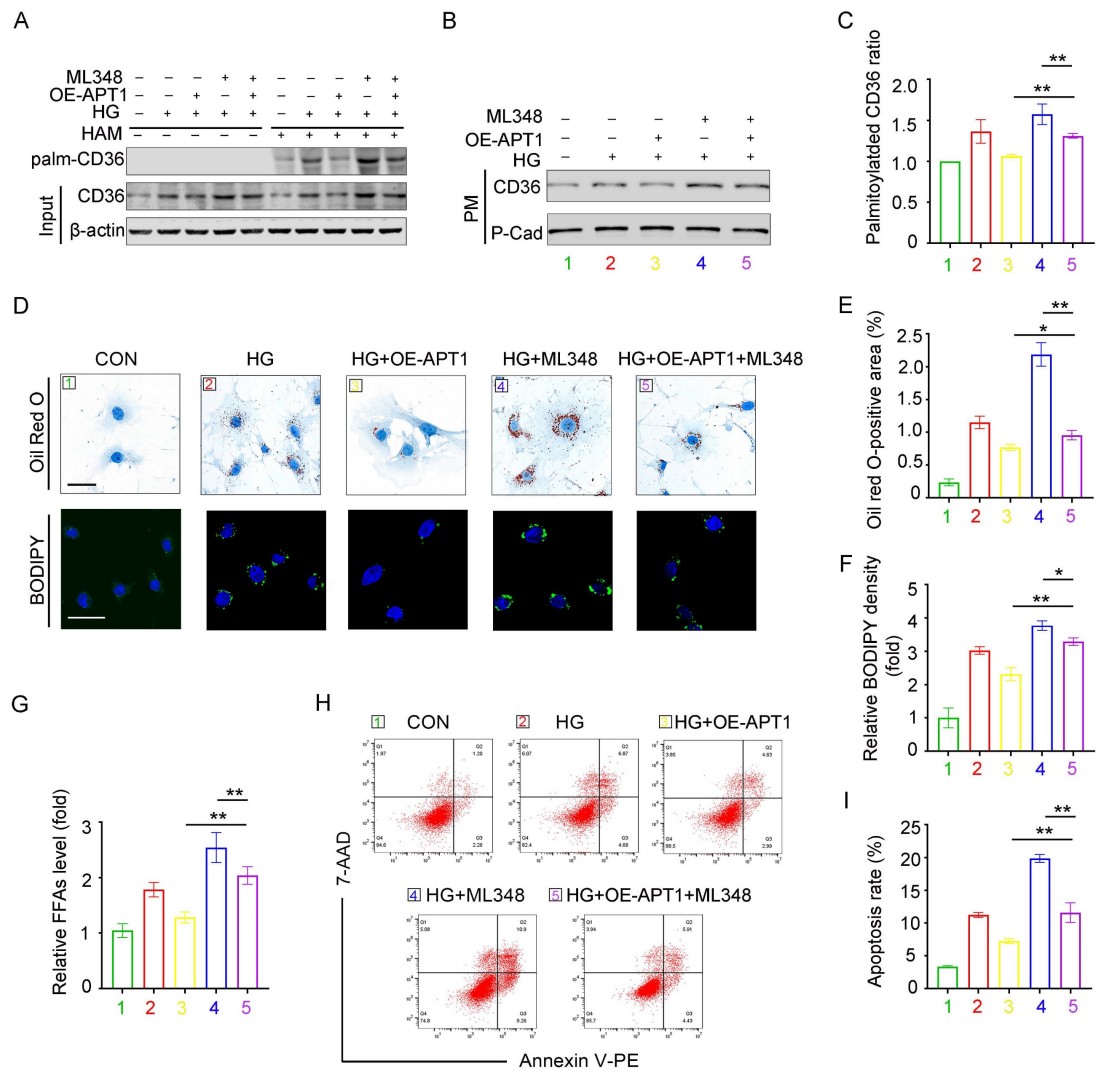
** APT1 inhibitor ML348 counteracted the change of APT1 plasmid in HPCs partially.** Podocytes were divided into five groups, (1) 5 mM normal glucose, (2) 30 mM high glucose for 24 hours, (3) APT1 overexpression plasmid transfection followed by incubation in the 30 mM high glucose for 24 hours, (4) 30 mM high glucose for 24 hours and 5 μM ML348 for 6 hours, (5) APT1 overexpression plasmid transfection followed by 30 mM high glucose for 24 hours and 5 μM ML348 for 6 hours. A-C. Representative images of palmitoylated CD36 (A), membrane-associated CD36 (B), and semiquantitative analysis (C). (**<0.01). D-F. Representative microscopy images of lipid droplets by Oil Red O (scale bar: 40 μm) and BODIPY staining (scale bar: 40 μm) (D), and semiquantitative analysis (E, F). (*<0.05, **<0.01). G. Cellular free fatty acids semiquantitative analysis. (**<0.01). H-I. Flow cytometry analysis of podocyte apoptosis and semiquantitative analysis. (**<0.01).

The APT1 inhibitor ML348 was administered to podocytes with or without APT1 overexpression plasmid transfection. The OE-APT1 + ML348 group counteracted the APT1 overexpression-induced decrease in palmitoylated, membrane-associated CD36 and total CD36 levels under HG stimulation (Figure 6A-C, Supplement 3A-C). Consistently, the OE-APT1 + ML348 group exhibited greater lipid accumulation than the OE-APT1 group, as shown by Oil Red O staining and BODIPY 493/503 (Figure 5D-F). Moreover, compared to the OE-APT1 group, the OE-APT1 + ML348 group had elevated intracellular FFA content and enhanced podocyte apoptosis (Figure 5G-I). These results indicate that ML348 can partially abolish the effects of APT1 on podocyte lipid metabolism.

### The effect of AAV-mediated APT1 overexpression in podocytes of DKD mice

**Figure 7:**
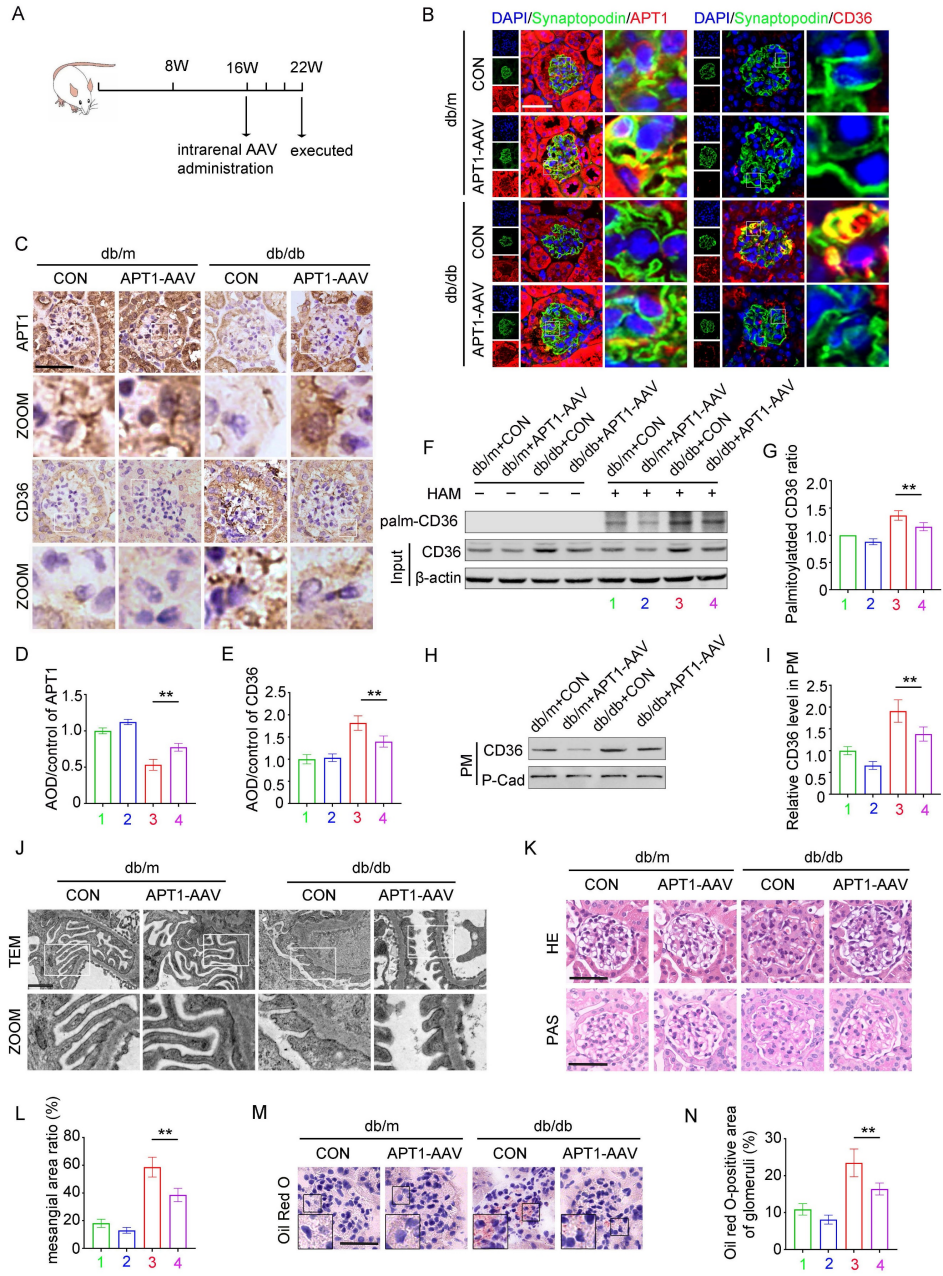
** The effect of APT1 overexpression on lipid metabolism in diabetic podocytes.** Mice were divided into four groups, (1) db/m+vector, (2) db/m+APT1-AAV, (3) db/db+vector, (4) db/db+APT1-AAV. A. Illustration of the animal model construction. B-E. Representative immunohistochemical (B) and immunofluorescence(C) staining of APT1 and CD36 in the glomeruli of each group, semiquantitative analysis (D,E). (**<0.01, scale bar: 50 μm). F-G. Representative images of palmitoylated CD36 (F) in each group, semiquantitative analysis(G). (**<0.01). H-I. Representative images of membrane-associated CD36 in each group, and semiquantitative analysis. (**<0.01). J. Ultrastructure of podocytes according to transmission electron microscopy in each group. (scale bar:10μm). K-L. Kidney pathology by HE and PAS staining and semiquantitative analysis. (**<0.01, scale bar:50μm). M-N. Representative images of Oil Red O staining in each group and semiquantitative analysis. (**<0.01, scale bar:50μm).

*In vivo*, the effects of APT1 overexpression on podocyte lipid metabolism were further validated. 16-week-old mice were intrarenally injected with adeno-associated virus (AAV) and sacrificed at 22 weeks of age (Figure 7A). Immunofluorescence, immunohistochemical staining, and Western blotting all verified that OE-APT1-AAV transfection significantly upregulated APT1 expression (Figure 7B-E, Supplement 4A-C). Consistent with *in vitro* results, OE-APT1-AAV transfection reduced total palmitoylated protein, palmitoylated CD36 ratio, and the membrane-associated CD36 level in the glomeruli (Figure 7F-I, Supplement 4D). Additionally, we observed a significant reduction in serum triglycerides, serum cholesterol (Supplement 4E-F), UACR (Supplement 4G) and serum creatinine in OE-APT1-AAV-transfected db/db mice compared to db/db controls (Supplement 4H). Notably, OE-APT1-AAV transduction markedly ameliorated diabetes-induced podocyte injury and glomerular damage, including mesangial proliferation and sclerosis, narrowing of peripheral capillary loops, foot process effacement, and glomerular basement membrane thickening (Figure 7J-L). Furthermore, lipid accumulation was significantly decreased in OE-APT1-AAV-transfected db/db mice compared to db/db controls (Figure 7M-N, Supplement 4J).

## Discussion

This study demonstrated that diabetes and HG stimulation downregulate APT1-dependent depalmitoylation of CD36, thereby enhancing CD36-mediated fatty acid uptake. APT1 was found to regulate the cellular recycling and degradation of CD36. Specifically, APT1 inhibition promotes CD36 palmitoylation, reduces its lysosomal degradation, and activates its recycling pathway, leading to increased CD36 enrichment on the plasma membrane, elevated lipid accumulation and subsequent podocyte injury.

Recent studies have detected significant renal lipid accumulation in patients with DKD and animal models, contributing to cytotoxicity and renal dysfunction 15,26. Podocyte injury is increasingly linked to lipotoxicity 6, yet the pathophysiological significance of fatty acid deposition in podocytes remains unclear. Our study confirmed that changes in CD36 palmitoylation in podocytes altered its subcellular localization, leading to lipid metabolism disorders in diabetic states. As previously shown, CD36, a transmembrane glycoprotein, plays a pivotal role in lipid uptake and utilization in diabetes, obesity, and other conditions 13,27,28. Increased CD36 expression is directly associated with ectopic lipid accumulation in the kidney, contributing to abnormal lipid metabolism in podocytes 16,29.

CD36 is subjected to post-translational modifications, such as glycosylation, ubiquitination, and palmitoylation, which can alter its subcellular localization and function 30. Notably, CD36 palmitoylation affects its intracellular localization, enhances membrane lipid raft trafficking, and modulates its biological function 30. Our findings revealed that HG stimulation increased both CD36 expression and palmitoylated CD36 anchoring in the plasma membrane. Consistent with our results, other studies showed that CD36 expression in adipocyte membranes increased in response to FFAs, where it was palmitoylated by ZDHHC4 and ZDHHC5, facilitating free fatty acids uptake 31. Subsequently, CD36 was depalmitoylated and internalized with the assistance of Caveolin-1 25.

Protein S-palmitoylation is a reversible process catalyzed by the zinc finger DHHC-type (ZDHHC) protein family, while depalmitoylation is mediated by enzymes such as acyl-protein thioesterases (APT1/APT2), palmitoyl protein thioesterases (PPT1/PPT2), and alpha/beta hydrolase domain-containing proteins (ABHD17A/B/C) 32,33. PPT1/2 are primarily localized within lysosomes 33,34, and ABHD17a/b/c are principally located on the plasma membrane 34,35. Previous research has excluded ABHD17 as a depalmitoylase for CD36 25. APT1, a key depalmitoylase, is expressed in the kidney, predominantly localized in the cytoplasm and on the plasma membrane 33. Although APT1's role has been studied in renal tubules 24, its function in podocytes has not been previously reported. Interestingly, a recent study in adipocytes has demonstrated that APT1 is capable of depalmitoylating CD36 25. Our study demonstrated that APT1 expression was decreased in diabetic podocytes. Our finding aligns with previous observations that APT1 activity in endothelial cells and islet β-cells is diminished in response to HG 21,31, and APT1-deficient mice exhibited angiopathy similar to diabetic angiopathy because of disrupted R-Ras trafficking 36. Meanwhile HG also promoted protein palmitoylation in pancreatic β cells 23, and similarly we observed an increase in palmitoylated proteins in diabetic mice. Furthermore, we identified binding sites between APT1 and CD36 amino acid residues and confirmed their interaction in podocytes through immunoprecipitation and confocal microscopy, representing the first such demonstration. Interestingly, we observed that the expression of renal tubule APT1 was also decreased in the diabetic mouse model, similar to that in mice with renal fibrosis due to unilateral ureteral obstruction (UUO) or ischemia reperfusion injury (IRI) 24. Since fatty acid oxidation is a crucial pathway for energy supply in renal tubular epithelial cells 37. It can be hypothesized that the upregulation of CD36, in conjunction with the downregulation of APT1, facilitates the translocation of CD36 to the renal tubular plasma membrane, thereby enhancing fatty acid uptake under conditions of impaired glucose utilization 38.

CD36, a membrane protein present on the cell surface, endoplasmic reticulum (ER), mitochondria, and Golgi apparatus, is transported between cellular organelles *via* the endosomal pathway 39. Our study is the first to demonstrate that Rab11a enhances CD36 recycling, facilitating its trafficking to the plasma membrane. Ligand-bound CD36 undergoes endocytosis to form early endosomes, which then face two possible fates: sorting into late endosomes and lysosomes for degradation or recycling back to the plasma membrane *via* recycling endosomes 40. However, the role of APT1 in regulating these pathways remains unclear. Thus, our findings reveal that under high glucose conditions, APT1 expression is decreased, leading to increased palmitoylated CD36 in the recycling pathway and reduced CD36 ubiquitination in the late endosome/lysosome pathway. In contrast, normal glucose conditions induce the opposite effect. In summary, the decision between degradation and recycling pathways for CD36 is primarily determined by its palmitoylation status. A similar mechanism has been observed with PD-L1, where palmitoylation inhibits ubiquitination, enhancing PD-L1 stability and preventing its degradation, thereby increasing its membrane expression 41,42. Conversely, in some cases, such as with the E3 ligase PHF2, palmitoylation triggers ubiquitination and subsequent degradation 43. Notably, CD36 not only facilitates fatty acid uptake from the extracellular environment but also mediates fatty acid transport between cells 44. Polarized endothelial cells regulate fatty acid uptake *via* transcytosis of CD36 and circulating fatty acids, subsequently exporting them to adjacent parenchymal cells through small exosome-like extracellular vesicles 44. Previous studies using CD36 mutant plasmids (CSSS, SCSS, SSCS, SSSC) transfected into HEK293 cells showed that the palmitoylation level of SSSC was closest to that of wild-type CD36 45. In hepatocytes, mutating the four palmitoylation sites of CD36 significantly affects its membrane localization 46, though the specific site with the most pronounced effect remained unidentified. This study is the first to establish that the Cys466 locus has the greatest impact on CD36 membrane localization in podocytes. Additionally, it has been reported that fatty acid overload exacerbates cell apoptosis and inflammation by activating the TLR4-NF-κB, NLRP3, JNK signaling pathways 25,47,48, which explains that lipotoxicity contributes to the apoptosis in HPCs in our study.

APT1 modulates CD36 trafficking and enhances the uptake of free fatty acids, thereby altering lipid droplet content. Given CD36's role in oxLDL and cholesterol accumulation, APT1 could also influence cholesterol levels in various organelles 49,50. Additionally, by affecting fatty acid availability, APT1 may indirectly impact phospholipid metabolism and the composition of organelle membrane phospholipids. Reductions in renal lipid accumulation, plasma triglyceride and cholesterol were observed in DKD mice with overexpression of APT1.

However, our study has several limitations. First, the major limitation is the absence of evidence for APT1's protective role in a transgenic mouse model. Second, it is unclear whether APT1 influences CD36 palmitoylation through changes in enzymatic activity. Third, given the lack of intrinsic enzymatic activity in CD36 49, APT1 may interact with CD36 through intermediate proteins to promote its internalization or activate downstream signaling pathways 13,25,49. Fourth, the transcriptional regulation of APT1 should be explored in future work. Based on available findings. we propose that APT1 is activated when it is palmitoylated or phosphorylated by Wnt5a 51, and that miR-138 regulates transcription of APT1 52. Additionally, Further studies incorporating human renal histological analysis are needed to enhance our understanding.

In summary, our study is the first to demonstrate that decreased APT1 and increased membrane palmitoylated CD36, regulated by the endosomal recycling pathway, lead to greater free fatty acid uptake and podocyte injury. Therefore, APT1's substantial physiological roles underscore its importance in metabolic diseases, and these findings offer a new direction for the development of novel therapeutic strategies 21. Future research should focus on developing small-molecule agents or biologics that specifically modulate APT1 activity and expression, potentially synergizing with existing therapeutic approaches to further enhance treatment efficacy in diabetic kidney disease.

## Materials and Methods

### Animal studies

Thirteen-week-old male db/db mice and age-matched db/m mice were purchased from Cavens Laboratory Animals (Jiangsu, China). At 16 weeks of age, the mice underwent intrarenal administration of adeno-associated virus (AAV) with titers of approximately 2.0x10^12^ viral genomes/ml. The AAV vector used was AAV9-CMV packaging either plasmid APT1(APT1-AAV) or a negative control (NC-AAV) (Hanbio, China).The mice were randomly divided into four groups: Group 1, db/m mice were injected with NC-AAV (n=5); Group 2, db/m mice were injected with APT1-AAV (n=5); Group 3, db/db mice were injected with NC-AAV (n=5); and Group 4, db/db mice were injected with APT1-AAV (n=5). Twenty-four-hour proteinuria, UACR, blood glucose, body weight were tested after injection. At 22 weeks of age, the kidneys and blood samples were harvested for histological and biochemical analyses. All animal experimental procedures were approved by the Ethics Committee for the Experimental Use of Animals of Renmin Hospital of Wuhan University.

### Cell culture and treatment

Conditionally immortalized human podocytes (HPCs) were kindly provided by Dr. Moin A. Saleem (Academic Renal Unit, Southmead Hospital, Bristol, UK). HPCs were cultured at 33°C for proliferation as previously described. Upon reaching 80% confluence, the podocytes were differentiated at 37°C for 7 days. Differentiated podocytes were synchronized for 24 hours in serum-free medium, followed by culture in various media (normal glucose, 5 mM glucose; mannitol, 5 mM glucose + 25 mM mannitol; high glucose, 30 mM glucose). ML348 (APT1 inhibitor, HY-100736, MCE) was administered at 5 μM, and MG132 (proteasome inhibitor, HY-13259, MCE) was added at 10 µM.

### Cell transfection

Plasmid transfection was performed using X-tremeGENE HP DNA Transfection Reagent (06366236001, Roche) according to the manufacturer's instructions. The expression plasmid pECMV-3xFlag-LYPLA1, encoding the full-length APT1 protein, was obtained from Miaolingbio (Wuhan, China). The expression plasmid pcDNA3.1-3xMyc-CD36-WT encoding the full-length CD36 protein (wild-type CD36), and mutant plasmids encoding site-directed CD36 variants (C3S, C7S, C464S, C466S) were constructed by Paivibio. Small interfering RNAs (siRNAs) targeting APT1, the lentiviral vector pLVXshRNA2-ZSGreen-T2A-Puro packaging shCD36(Lv-shCD36) or the negative control (Lv-CTL) were also sourced from Paivibio.

### Free fatty acids and triglycerides quantification assay

Free fatty acid detection in cells was processed using the Amplex Red Free Fatty Acid Test Kit (S0215S, Beyotime, China).

### Oil red O staining assay

To measure total neutral lipid accumulation in kidney tissue, frozen kidney sections were cut into 8 µm slices and stained with Oil Red O (G1015, Servicebio). Podocytes cultured on glass slides were fixed in 4% paraformaldehyde for 30 minutes at 4°C, rinsed in Phosphate Buffered Saline (PBS), equilibrated in 60% isopropanol for 30 seconds at room temperature, and then incubated in 0.5% Oil Red O for 30 minutes. After a brief rinse with 60% isopropanol for 1 minute to differentiate the staining, the cells were rinsed in PBS, counterstained with hematoxylin for 1 minute, and mounted onto glass slides. An Olympus microscope (Olympus, Japan) was used to visualize the Oil Red O-stained sections.

### BODIPY 493/503 fluorescent staining

For staining neutral lipids in the cytoplasm, BODIPY 493/503 (a standard fluorescent dye) was used. Cell slides were fixed with 4% paraformaldehyde, and stained with BODIPY 493/503 (4,4-difluoro-1,3,5,7,8-pentamethyl-4-bora-3a,4a-diaza-s-indacene, 1 μM, D3922, ThermoFisher, USA) in the dark for 1 hour. After discarding the dye, the cells were counterstained with DAPI fluoromount (ANT063, China) for 15 minutes, dried, and sealed. The slides were then observed under the Olympus confocal fluorescence microscope (Olympus, Japan). Frozen kidney sections were fixed, blocked, and then stained with WT1 antibody (BM4216, Boster, China) for 1 h at 37℃, subsequently incubated in the presence of secondary antibody and BODIPY dye, and finally covered with DAPI.

### Western blotting analysis

For Western blotting analysis, total proteins were extracted from glomeruli or HPCs using lysis buffer (P0013, Beyotime, China) supplemented with protease inhibitor cocktail (HY-K0010, MCE, USA), phosphatase inhibitor cocktail (HY-K0013, MCE, USA), and PMSF (ST506, Beyotime, China). The extracts were centrifuged. Protein concentrations were determined using a BCA protein assay (P0010S, Beyotime, China), and then boiled with a loading buffer. Electrophoresis and electroblotting were performed, and then the samples were incubated with primary and secondary antibody. The membranes were then treated with enhanced chemiluminescence solution (ECL, G2014, Servicebio, Wuhan, China) and visualized using an X-ray machine (Bio-Rad, USA). The primary antibodies are as follows: anti-CD36 (mouse 1:500, sc-7309, Santa Cruz, USA), anti-APT1 (rabbit 1:1000, 16055, Proteintech, China), anti-rab11a (rabbit 1:5000, 71-5300, Invitrogen), anti-β-actin (mouse 1:5000, 60008, Proteintech, China), anti-flag-tag (rabbit 1:1000, PM020, MBL), anti-myc-tag (mouse 1:5000, M192-3, MBL). The secondary antibodies are as follows: HRP-conjugated secondary antibodies (ANT019, ANT020, 1:10000, Antgene, Wuhan, China).

### Immunohistochemistry

Immunohistochemistry was conducted as previously described, including antigen retrieval. After blocking, the slides were incubated overnight with primary antibodies (mouse CD36, 1:200, sc-7309 Santa Cruz; rabbit APT1, 1:200, 16055, Proteintech). HRP-conjugated goat anti-mouse/rabbit IgG secondary antibody was then applied. Images were captured using an upright microscope (Olympus, Japan).

### Immunofluorescence

For immunofluorescence, sections or cell slides were incubated with primary antibodies (mouse CD36, 1:200, sc-7309, Santa Cruz; APT1, 1:200, 16055, Proteintech; Synaptopodin, 1:100, sc-515842, Santa Cruz; rabbit CD36, 1:200, NB400-144, Novus; mouse Lamp1, 1:200, sc-20011, Santa Cruz; Rabbit Rab7a, 1:200, 55469 Proteintech; Rabbit Rab11a, 1:200, 71-5300, Invitrogen), and then followed by fluorescent secondary antibodies (anti-Mouse 488 ANT023s, anti-Rabbit 594 ANT030s, anti-Mouse 594 ANT029s, anti-Rabbit 488 ANT024s, 1:100, Antgene, Wuhan, China). Nuclei were counterstained with DAPI (ANT063, Antgene, Wuhan, China). Images were also captured using an upright microscope (Olympus, Japan) or confocal microscopy (Leica Application Suite, Germany).

### Co-immunoprecipitation (CO-IP) and immunoprecipitation

Whole-cell extracts were prepared after treatment and incubated overnight at 4°C with 5 μl of primary antibodies and 30 μl of protein beads (protein L-Agarose Sc-2336, Santa Cruz; normal mouse IgM Sc-3881 Sant Cruze; L-1302 anti-myc beads, L-1303 anti-flag beads, L-1204 Protein A/G beads, Bio-Link, China; 101241 Recombinant IgG beads Invitrogen). The beads were washed three times on ice, then boiled in loading buffer at 100°C for 10 minutes before SDS-PAGE. The subsequent steps followed standard Western blotting procedures.

### Transmission electron microscopic analysis

Kidneys were isolated and fixed in 4% paraformaldehyde for 30 minutes at room temperature. Sections were stained with periodic acid-silver methenamine. As previously described, ultrastructural changes in renal tissues fixed in 2.5% glutaraldehyde were examined using transmission electron microscopy (TEM, Hitachi, Japan).

### Isolation of cellular plasma membrane fractions

The plasma membrane-rich fraction was isolated using the Mem-PER Plus Membrane Protein Extraction Kit (89842, Thermo Fisher, USA). Briefly, cells were harvested from 100 mm dishes, centrifuged, and washed twice. The cell pellet was treated with permeabilization buffer, vortexed, incubated, and then centrifuged. The supernatant containing cytosolic proteins was separated, while the pellet was mixed with solubilization buffer, followed by incubation, vortexing, and centrifugation. The resulting supernatant, containing solubilized membrane and membrane-associated proteins, was stored at -80°C for future analysis.

### Acyl-biotin exchange (ABE) assay

The ABE assay was performed according to the protocol of the IP-ABE Palmitoylation Kit (AM10314, AIMS, China). Proteins from HPCs and kidney tissues were extracted in lysis buffer with a protease inhibitor cocktail. Antigen-antibody complexes were obtained with A/G magnetic beads through immunoprecipitation. The ABE procedure involved blocking, reduction, labeling, elution, and detection. Tris(2-carboxyethyl)phosphine (TCEP) was used to reduce disulfide bonds, and N-ethylmaleimide (NEM) was used to block unmodified cysteines for 30 minutes. The beads were then washed and divided, with one part treated with hydroxylamine (HAM) and the other left untreated. Both were incubated for 1 hour at room temperature. After washing, the beads were incubated with thiol-reactive biotin for 1 hour at room temperature. The samples were collected with a loading buffer and analyzed by Western blotting. The ABE assay of total proteins was conducted following the protocol of the ABE Palmitoylation Kit (AM10316, AIMS, China).

### Flow cytometry analysis

Cell apoptosis was measured using the Annexin V Apoptosis Detection Kit I (559763, BD Biosciences). Cells were digested with trypsin, neutralized, centrifuged, and resuspended in 100 μl of 1× binding buffer. Each sample was transferred to a 5 ml tube, where 5 μl of 7-ADD and 5 μl of PE Annexin V were added, followed by 15 minutes of incubation at room temperature in the dark. After resuspension, the cells were analyzed using a Cytoflex flow cytometer (Beckman).

### Statistical analysis

Experiments were conducted with a minimum of three replicates per condition. Data are presented as the mean ± standard deviation. Statistical significance (p < 0.05) was assessed using Student's t-test or one-way ANOVA, with analyses performed in GraphPad Prism 7 (GraphPad Software, Inc., La Jolla, CA).

## Supplementary Material

Supplementary figures.

## Abbreviations

AAV: adeno-associated virus
ABE: acyl-biotinyl exchange
UACR: urine albumin creatinine ratio
APT1: acyl-protein thioesterase 1
ANOVA: analysis of variance
CD36: Cluster of Differentiation 36
CKD: chronic kidney disease
DKD: diabetic kidney disease
ER: endoplasmic reticulum
ESRD: end-stage renal disease
FFA: free fatty acid
HAM: hydroxylamine
HG: high glucose
HPCs: human podocytes
LDs: lipid droplets
IP: immunoprecipitation
OE: overexpression
oxLDL: oxidized low-density lipoprotein
PAS: periodic acid-schiff stain
PBS: Phosphate Buffered Saline
TEM: transmission electron microscope
WCL: whole cell lysate
WT: wild type
WT1: wilms' tumor gene 1
