# Supplementary material for: APT1-derived depalmitoylation of CD36 alleviates diabetes-induced lipotoxicity in podocytes
Source: Int J Biol Sci. 2025 Jun 9;21(9):3852–66. doi: 10.7150/ijbs.109220 (PMC12210380; doi:10.7150/ijbs.109220)

Supplement 1

A-B. The alterations of CD36 on the plasma membrane and in the whole cell lysate (WCL) under normal glucose, and high glucose (30 mM) conditions in the presence of a glycation inhibitor (Tun, 5 μmol/L, 12 hours, MCE HY-A0098), a ubiquitination inhibitor (TAK-243, 500 nmol/L, 6 hours, HY-100487), a phosphorylation inhibitor (K-252a, 50 nmol/L, 24 hours, MCE HY-N6732), and a palmitoylation inhibitor (2-BP, 100 μmol/L, 12 hours, MCE, HY-111770).Tun: tunicamycin, 2-BP: 2-Bromohexadecanoic acid, Representative Western blot of CD36 in each group, and semiquantitative analysis (*<0.05, **<0.01).

C. Immunoprecipitated by anti-flag antibody, the precipitates and a part of whole cell lysate were subjected to immunoblotting with anti-myc and anti-flag antibodies.

Supplement 2

Podocytes were transfected with empty vector or APT1 plasmid, then incubated with or without 30 mM high glucose for 24 hours.

A-C. Representative Western blot of APT1 and CD36 in each group of HPCs with or without APT1 plasmid under normal glucose or high glucose(D), and semiquantitative analysis (E, F). (*<0.05, **<0.01).

D-E. CO-IP with anti-CD36 antibody or an IgM negative control (A), CO-IP with anti-Rab11a antibody or an IgG negative control (B).

F. Ubiquitinated CD36 in HPCs with or without APT1 overexpression plasmid incubated with 30 mM HG stimulation for 24 hours and 10 μM MG132 for 6 hours.

Supplement 3

Podocytes were divided into five groups, (1) 5 mM normal glucose, (2) 30 mM high glucose for 24 hours, (3) APT1 overexpression plasmid transfection followed by incubation in the 30 mM high glucose for 24 hours, (4) 30 mM high glucose for 24 hours and 5 μM ML348 for 6 hours, (5) APT1 overexpression plasmid transfection followed by 30 mM high glucose for 24 hours and 5 μM ML348 for 6 hours.

A-C. Representative Western blot of APT1 and CD36 in each group of HPCs and semiquantitative analysis. (*<0.05, **<0.01).

Supplement 4

A-C. Representative western blot of APT1 and CD36 in the glomeruli of each group, and semiquantitative analysis. (*<0.05, **<0.01).

D. Palmitoylation level of total protein in the glomeruli of each group by ABE method and Western blot.

E-I. Biochemical tests in each group: serum triglycerides (E) , serum cholesterol (F), UACR (G), serum creatinine (H), blood glucose (I). (*<0.05, **<0.01).

J. Representative immunofluorescence double staining of BODIPY and WT1 in the glomeruli of each group, and semiquantitative analysis. (scale bar:40μm).

Supplement 1

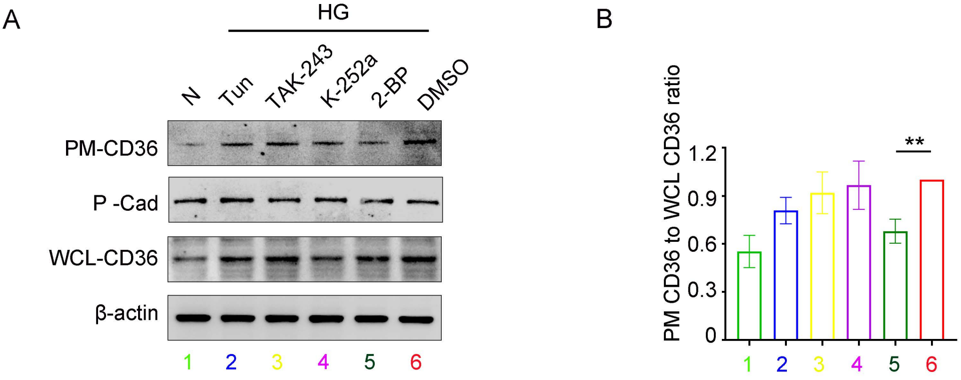

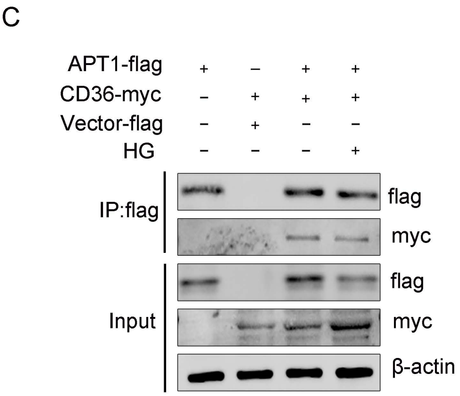

Supplement2

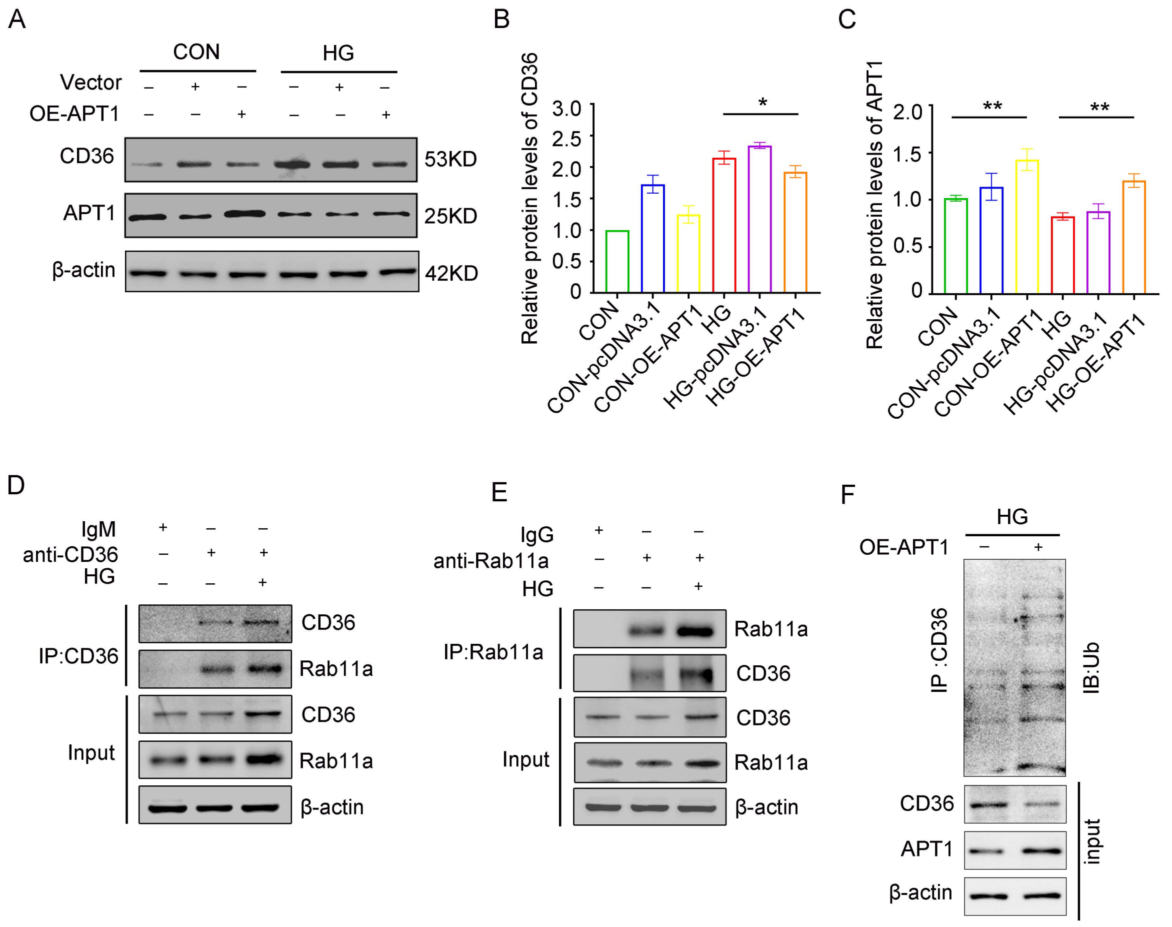

Supplement3

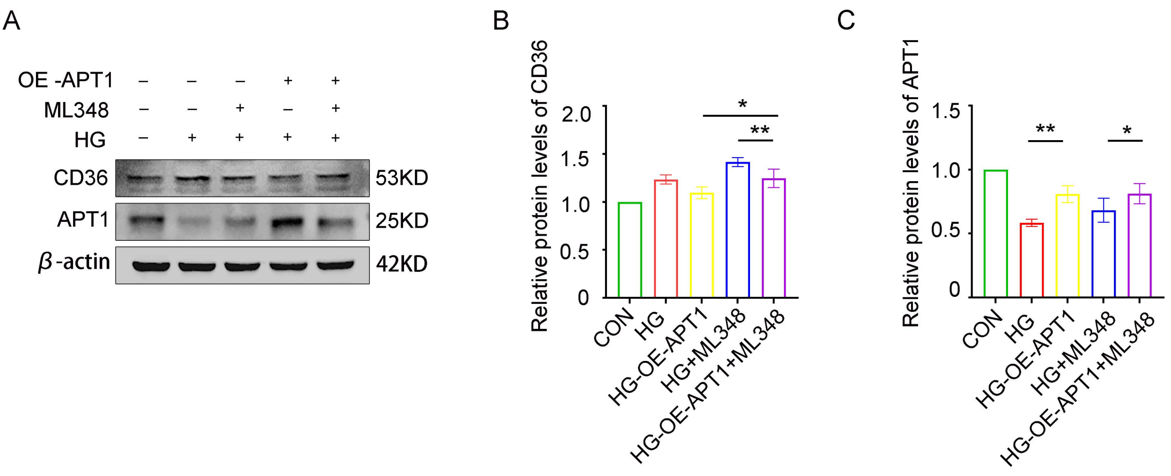

Supplement4

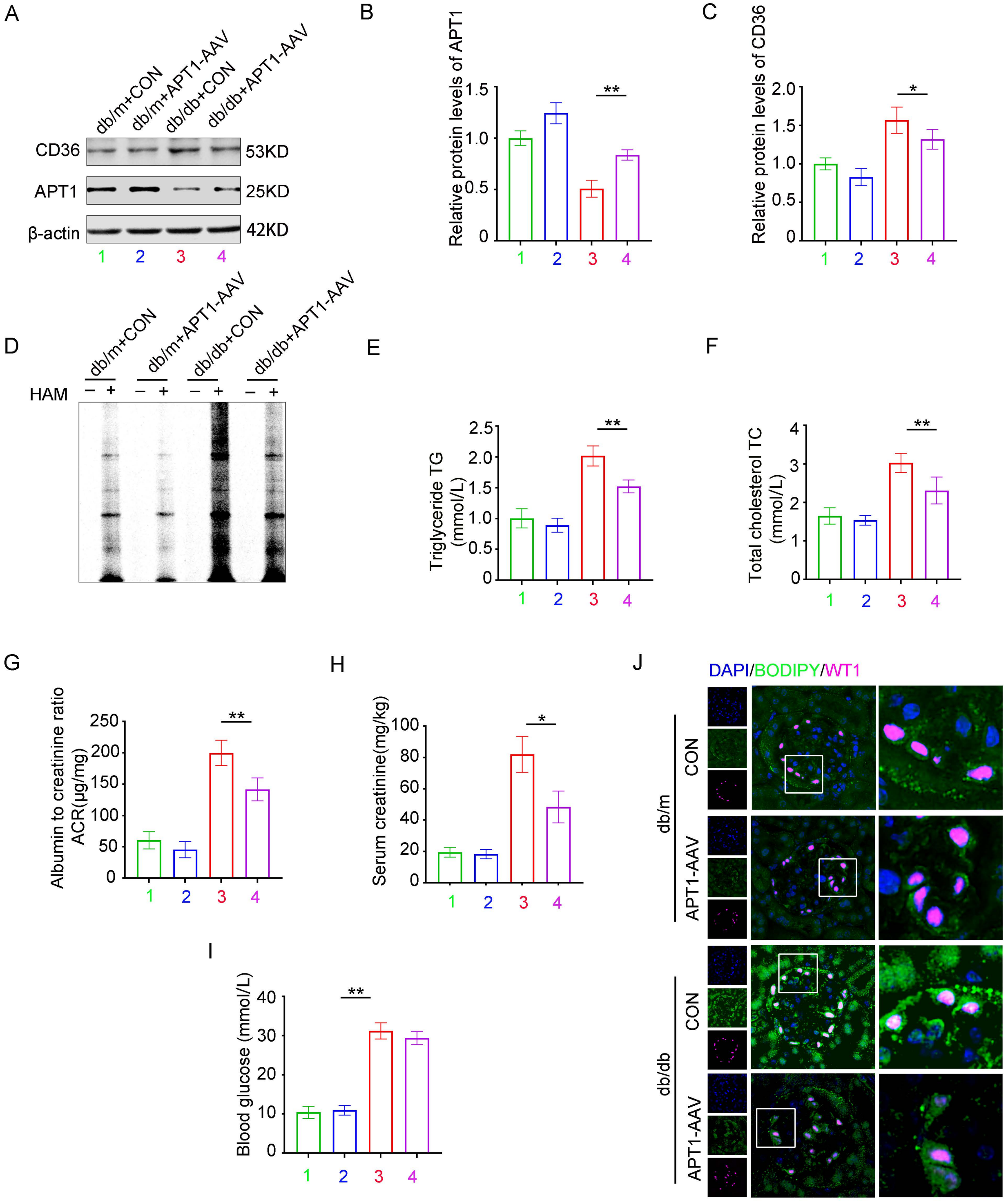

Supplement: Supplementary file 1 — Supplementary figures. [file ijbsv21p3852s1.pdf]
